# Endurance exercise upregulates *mtp* expression in aged *Drosophila* to ameliorate age‐related diastolic dysfunction and extend lifespan

**DOI:** 10.14814/phy2.15929

**Published:** 2024-02-02

**Authors:** Tianhang Peng, Meng Ding, Hanhui Yan, Ping Zhang, Rui Tian, Yin Guo, Lan Zheng

**Affiliations:** ^1^ Key Laboratory of Physical Fitness and Exercise Rehabilitation of Hunan Province Hunan Normal University Changsha China

**Keywords:** aging, cardiac function, *Drosophila*, endurance exercise, lifespan, *mtp*

## Abstract

Diastolic dysfunction is a major cardiac dysfunction, and an important predisposing factor is age. Although exercise training is often used for the prevention and treatment of cardiovascular disease nowadays, little is currently known about whether exercise interventions associated with the slowing of cardiac aging are related to *mtp*‐related pathways. In the present study, the UAS/Tub‐Gal4 system was used to knockdown whole‐body *mtp* expression levels in *Drosophila*, which underwent 2 weeks of endurance training. By conducting different assays and quantifying different indicators, we sought to investigate the relationship between *mtp*, exercise, and age‐related diastolic dysfunction. We found that (1) *Drosophila* in the *mtp* ^*RNAi*^ youth group exhibited age‐related diastolic dysfunction and had a significantly shorter mean lifespan. (2) Endurance exercise could improve diastolic dysfunction and prolong lifespan in aged *Drosophila*. (3) Endurance exercise could increase the expression levels of *apolpp* and *Acox3*, and decrease the levels of TC, LDL‐C, and TG in the aged group. In summary, aging causes age‐associated diastolic dysfunction in *Drosophila*, and systemic knockdown of *mtp* causes premature age‐associated diastolic dysfunction in young *Drosophila.* Besides, endurance exercise improves age‐related diastolic dysfunction and prolongs lifespan.

## Graphical abstract

Endurance exercise ameliorates age‐related diastolic dysfunction and prolongs average lifespan, which may be related to the upregulation of mtp expression and thus enhance lipid metabolism in aged *Drosophila*.

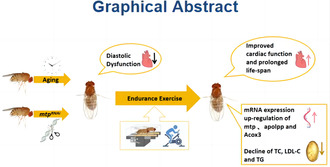

## 1 INTRODUCTION

Cardiovascular health is closely related to longevity, and cardiovascular disease (CVD) represents the leading cause of death in people aged 65 years and older. Diastolic dysfunction is a major cardiac dysfunction that can lead to diastolic heart failure, which impairs myocardial contractility as well as pumping capacity in the elderly, and an important predisposing factor is age (Kass et al., 2004). Aging, as a primary risk factor for cardiovascular disease, lowers the threshold for cardiovascular disease by promoting adverse changes in cardiac structure and function, with significant effects on the heart and vasculature, including atherosclerosis, hypertension, myocardial infarction, and stroke. Cardiac aging is associated with left ventricular remodeling, characterized by increased mass‐to‐volume ratio (Lakatta & Levy, 2003; North & Sinclair, 2012). The molecular mechanisms regulating cardiac aging remain incompletely understood, warranting further research.

The past few years have witnessed unprecedented progress in research on longevity genes. Microsomal triglyceride transfer protein (*mtp*), a direct homolog of human MTTP (microsomal triglyceride transfer protein), is a lipid transfer protein found in the liver and intestine. Given its role as a rate‐limiting enzyme in lipid metabolism, MTP has been associated with human longevity, coronary artery disease, and other vascular diseases caused by adverse lipid profiles (peripheral vascular disease, renal vascular disease, and stroke), which account for a significant portion of human mortality (Geesaman et al., 2003). Although *mtp* has been associated with many diseases, the link between *mtp* and age‐related diastolic dysfunction has not been elucidated.

Exercise is a fundamental health behavior that can prevent systemic cardiovascular disease and improve cardiovascular health through nontraditional mechanisms. Besides, it has been established that exercise and associated high levels of cardiorespiratory fitness can reduce cardiovascular mortality, the risk of heart failure and myocardial infarction, and age‐related arterial and cardiac stiffness. On the contrary, physical inactivity is a direct cause of organismal aging and a recognized risk factor for cardiovascular disease. Over the years, many epidemiological studies have reported that low activity and sedentary behavior lead to a 63% increase in the risk of developing cardiovascular disease (Jakovljevic, 2018). Accordingly, physical activity and exercise can attenuate age‐related cardiovascular changes by improving cardiovascular system function and metabolism. It has been reported that moderate‐intensity (≤70% of VOmax) dynamic exercise, which primarily involves aerobic energy pathways and large muscle mass (e.g., brisk walking and cycling), attenuates age‐related declines in cardiorespiratory fitness (Moore et al., 2012). In addition, elite athletes who maintain very high activity levels (e.g., former participants in the Tour de France or former Olympic marathon runners) typically exhibit greater longevity than the general population (Garatachea et al., 2014). Exercise to slow aging and reduce the risk of developing cardiovascular disease has been a hot topic of interest in medical and exercise science research, but it remains uncertain whether exercise amelioration of aging‐induced cardiovascular disease is linked to *mtp* and its molecular pathways.

The fruit fly *Drosophila melanogaster* has been shown to be an effective model for studying aging and adult cardiac function (Hughes & Reynolds, 2005; Piazza & Wessells, 2011). The *Drosophila* heart or dorsal blood vessel is a linear tube reminiscent of the primitive vertebrate embryonic heart tube, and although the final cardiac structure of *Drosophila* is very different from that of the vertebrate heart, the basic elements of cardiac development, function, and aging are very well conserved (Bodmer, 1995). The *Drosophila* heart model has proven to be an invaluable asset in elucidating the etiology of human cardiac disease, including dilated and restrictive cardiomyopathies, ion channelopathies, diabetic and congenital heart disease, and cardiac aging (Cammarato et al., 2008; Taghli‐Lamallem et al., 2008; Wessells et al., 2004). The *Drosophila* heart has also been used to identify new genes that may be involved in heart disease (Qian et al., 2011).

In the present study, after establishing a *Drosophila* exercise model, we found that cardiac systolic function and *mtp* expression levels decline with aging in *Drosophila*. Besides, there is a strong association between age‐related diastolic dysfunction and *mtp* expression levels. Importantly, endurance exercise improves age‐related diastolic dysfunction and prolongs lifespan, possibly related to the upregulation of *mtp* expression, thereby enhancing lipid metabolism in aged *Drosophila*.

## 2 MATERIALS AND METHODS

### 2.1 *Drosophila* strains and culture protocol

*W* ^*1118*^ wild‐type, tub‐Gal4 regulatory line (preserved by Hunan Key Laboratory of Physical Fitness and Sports Rehabilitation, Hunan Normal University, Hunan Province, China). TH02325.N (UAS‐*mtp*RNAi) was obtained from the *Drosophila* Centre, Tsinghua University. All *Drosophila* were reared using a standard medium (made from a combination of yeast, corn, and starch).

### 2.2 *Drosophila* hybridisation and grouping

*W* ^*1118*^ and TH02325.N virgin flies were crossed with tub‐Gal4 males, and the resulting virgin flies of F1 generation were randomly divided into the *mtp* normal expression group (Tub × *W* ^*1118*^) and the *mtp* systemic knockdown group (*mtp* ^*RNAi*^). Virgin flies were selected as experimental subjects since female flies store more triglycerides than males, and female flies are larger and easier to observe and dissect. The *mtp* normal expression group was further divided into a 7‐day‐old normal intervention group (7d‐N), a 14‐day‐old normal intervention group (14d‐N), a 21‐day‐old normal intervention group (21d‐N), a 28‐day‐old group (28d‐N), a 35‐day‐old normal intervention group (35d‐N), and 35‐day‐old exercise intervention group (35d‐E). The *mtp* systemic knockdown group was subdivided into a 14‐day‐old normal intervention group (14d‐N), a 35‐day‐old normal intervention group (35d‐N), and a 35‐day‐old exercise intervention group (35d‐E). Normal intervention is denoted by N, and exercise intervention is denoted by E, where 14‐day‐old (14d) *Drosophila* represented the young group and 35‐day‐old (35d) *Drosophila* represented the old group. The F1 generation produced by the two major groups was used as the experimental *Drosophila* 800 each, 20 flies/tube, and all *Drosophila* were put into the HWS intelligent constant temperature and humidity incubator, with a constant temperature of 25, constant humidity of 50%, and a 12‐h day/night cycle of rearing. The intervention was carried out on the 22nd day after *Drosophila* fledged in the old age group, where sampling was carried out on day 36, while sampling was carried out on day 15 after *Drosophila* fledged in the young age group.

### 2.3 Training programme

We used a *Drosophila* locomotor activity monitor to simulate the effects of locomotion on aging phenotypes. The monitor was designed to induce upward walking by taking advantage of the innate negative geotactic behavior of *Drosophila*. Flies were aged to 22 days post‐eclosion and exercised in vials with an inner diameter of 2.8 cm, rotating at 0.16 rpm,1.5 h per exercise for 2 weeks, with 5 days of training and 2 days of rest planned. Exercise sessions were conducted from 14:00 to 15:30. Most *Drosophila* responded consistently by climbing throughout the exercise period, and the few *Drosophila* that failed to climb were observed walking vigorously on the inner wall of the vials, which we termed as endurance exercise (Tinkerhess et al., 2012; Zheng et al., 2015).

### 2.4 Reverse transcription polymerase chain reaction

Ten flies were placed into 1 mL of Trizol reagent lysis solution (Invitrogen) for homogenization and RNA extraction. Trizol was used to extract the organic solvent, and 10 μg total RNA was purified using oligo (dT) synthesized from total RNA with superscript II reverse transcriptase (Invitrogen). qPCR amplification reactions were performed in triplicates by mixing 1 μL of RT product with 10 μL of SYBR qPCR Mastermix (TaKaRa) containing the appropriate PCR primers. Thermal cycling and fluorescence monitoring were performed in an ABI7300 (Applied Biosystems, United States) using the following PCR conditions: (30 s at 95°C, 5 s at 95°C, and 30 s at 60°C) × 40. Values were normalized with *rp49*. Primer purification was performed using polyacrylamide gel electrophoresis (PAGE). The primers (obtained from Beijing Tsingke Biotech Co., Ltd.) used were as follows:

rp49
  F: 5′‐CTAAGCTAGTCGCACAAATAGG‐3′
  R: 5′‐AACTTCTTAGAATCCGGTAGGG‐3′
mtp
  F:5′‐ACGGAAATCCAGCAGAACACT‐3′
  R:5′‐ATACGTAAAGCCAACGGCCA‐3′
Acox3
  F: 5′‐ACTTCCGTAGCGGACCTTTAG‐3′
  R: 5′‐GCAGAAGATAGTAGGGGGTTCCA‐3′
apoLpp
  F: 5′‐AATTCGC GGATGGTCTGTGTGT‐3′
  R: 5′‐GCCCCTTAGGGATA GCCTTT‐3′

### 2.5 Semi‐integrated *Drosophila* heart preparation and heartbeat analysis

After anesthetizing *Drosophila*, the head and ventral thorax were quickly removed and poured into oxygenated artificial haemolymph (AH), followed by the removal of the ventral abdominal cuticle and all viscera to expose the heart tube (Vogler & Ocorr, 2009). A 30‐s digital movie of high‐speed heartbeats was taken at 130 fps using an EM‐CCD high‐speed camera and recorded using HCImage software (Hamamatsu, Japan). Indicators of cardiac function, for example, heart period (HP), arrhythmia index (AI), diastolic interval (DI), systolic interval (SI), diastolic diameter (DD), systolic diameter (SD), fractional shortening (FS), and fibrillation (FL), were quantified using semiautomatic optical heartbeat analysis software (Fink et al., 2009).

### 2.6 Climbing test

The negative geotropic climbing ability test was adapted from a previously documented method (Coulom & Birman, 2004). The climbing device consisted of five 20 cm long glass tubes with an inner diameter of 2.8 cm (sponges are placed at the ends of the tubes to prevent escape but allow air exchange). The sponge plugs at the ends of the long glass tubes were 2 cm each, providing 16 cm of climbing space for the fruit flies. The long glass tubes were divided equally from bottom to top into 1, 2, 3, and 4 quadrants of 4 cm each. The *drosophila* flies were allowed to acclimatize to the vials for 30 min before their climbing ability was assessed. Negative geotropism was triggered by jolting the fruit flies to the bottom of the vials by rapidly and continuously vibrating the climbing device. The position of the fruit fly was taken from a digital image taken at the end of 10 s after the triggering behavior. This process was repeated five times with 20 flies per tube. These photographs were placed in Photoshop to analyze the climbing index, which was calculated using the following formula: Climbing index = number of fruit flies in quadrant 4/the total number of fruit flies in a glass jar.

### 2.7 Lifespan assay

Lifespan assays were carried out as described in a previous study (Zheng et al., 2015). Dead *Drosophila* were counted every 2 days. Ten replicates (approximately 200 *Drosophila*) were used per condition, and female *Drosophila* were used for all longevity experiments. Survival curves were plotted using GraphPad Prism software (San Diego, CA).

### 2.8 Protein blot analysis

A sample size of 10 *Drosophila* per group was homogenized in urea lysis buffer (PBS, pH 7.4, 5.0 M urea, 2.0 M thiourea, 50 mM DTT, 0.1% SDS) using a pestle and mortar and a sonicator at 4°C. The mixture was centrifuged at 12,000 g for 10 min at 4°C, and the resulting supernatant was used for protein blot analysis. Proteins were extracted from *Drosophila* and separated on a 10% SDS‐polyacrylamide (29:1) mini‐gel (Bio‐Rad) and subsequently electroblotted on a polyvinylidene difluoride membrane (0.45um). Primary antibodies were directed against microsomal triglyceride transfer protein (rat monoclonal anti‐myosin, ab75316, 1:500, Abcam, Massachusetts, USA). The secondary antibody was peroxidase‐coupled anti‐rat IgG (1:5000, Sigma). Proteins were visualized using Super Signal West Dura detection reagent (Thermo Scientific), and signals were detected using the ChemiGenius Bioimaging System. Analyses and quantitative assessments were performed using GraphPad Prism software (San Diego, CA). Experiments were performed in triplicate.

### 2.9 Cholesterol, low‐density lipoprotein, and triglyceride determination

Total cholesterol (TC), low‐density lipoprotein (LDL‐C), and triglyceride (TG) were determined using the corresponding assay kits (Nanjing Jiancheng Bioengineering Institution, China) according to the manufacturer's instructions (Diop et al., 2017). TC was measured at 500 nm, LDL‐C at 600 nm, and TG at 510 nm. The absorbance was measured at different wavelengths with a microplate reader (Shenzhen Huisong Technology Development Co., Ltd.), with the optical density (OD) value of the measured standard as the coordinate and the concentration of the standard as the ordinate. The standard curve was drawn on the coordinate paper or with relevant software, and the linear regression equation was obtained. The OD value of the sample was replaced in the equation to calculate the concentration of the sample. Ten flies were tested for each indicator.

### 2.10 Ghost pen cyclic peptide immunofluorescence staining

Semi‐intact *Drosophila* hearts were prepared and confirmed to show rhythmic beating in oxygenated ADH. ADH was quickly replaced by a relaxation buffer (containing 10 mM EGTA). The cardiac tissues were fixed in 4% formaldehyde for 20 min at room temperature, then washed three times (10 min per wash) with PBS at room temperature, and were stained with ghost cyclin (ghost cyclin‐iFluor 594,1:1000, Abcam) for 40 min followed by three washes with PBS at room temperature (10 min per wash). Fluorescence staining images were obtained with a confocal laser scanning microscope (Carl Zeiss; Oberkochen; Germany).

### 2.11 Statistical analysis

The data obtained were processed statistically using the Statistical Package for the Social Sciences for Windows (SPSS) version 24.0 (SPSSInc., Chicago, IL, USA), and the Shapiro–Wilk test was used to test the normal distribution of the samples in each group. If the data followed a normal distribution, we employed independent samples *t*‐test or ANOVA for analysis of variance. The mean ± standard deviation (SD) values are presented in figures for statistical descriptions. In cases where normal distribution assumptions were not met, the Kruskal‐Wallis H rank‐sum test was utilized. Data were analyzed using median and quartiles (M, P25, P75) for statistical description. The significance level was set at *p* < 0.05.

## 3 RESULTS

### 3.1 mtp knockdown induces age‐related diastolic dysfunction in young Drosophila similar to that in old Drosophila

**FIGURE 1:**
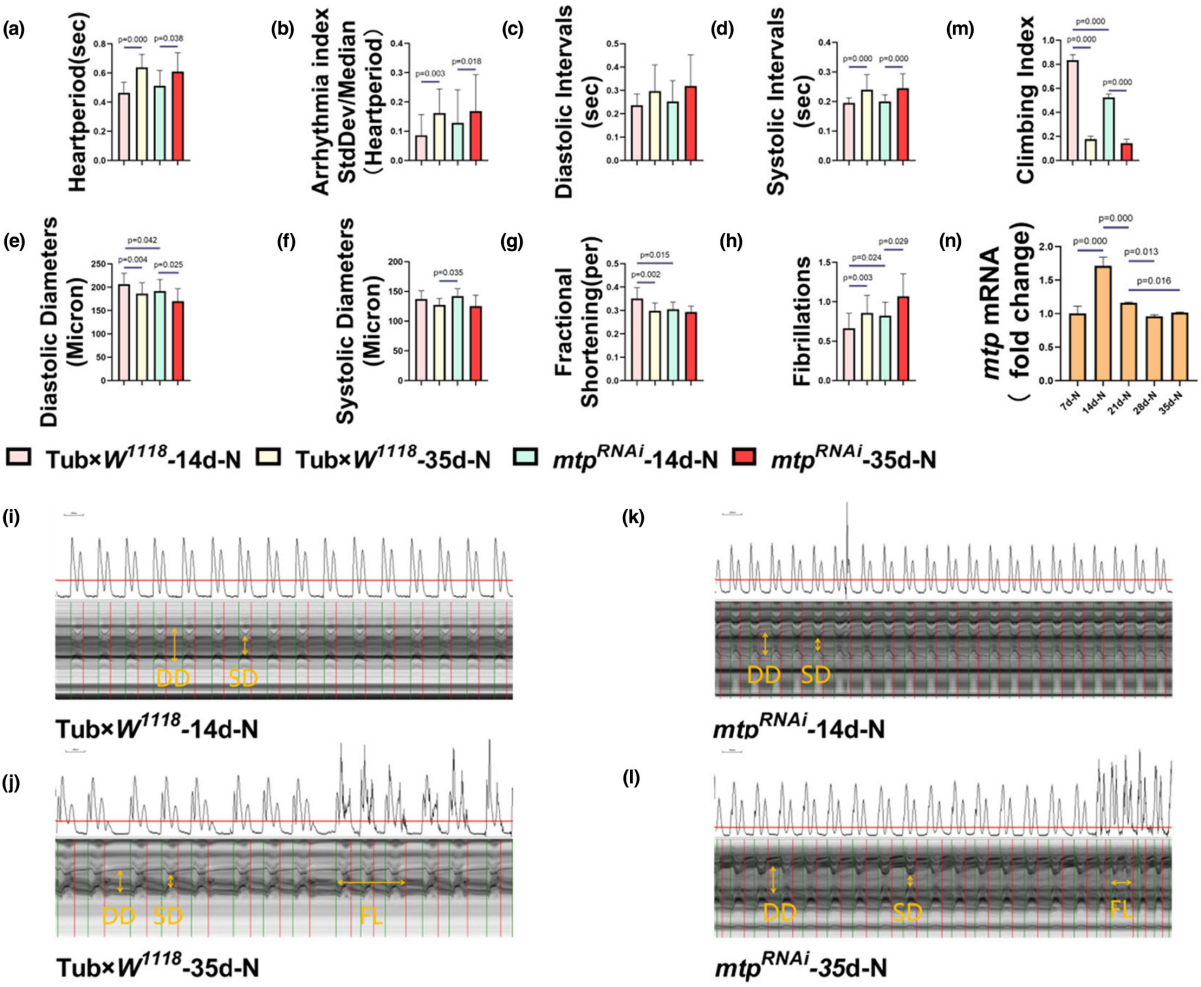
(a–h) the M‐type electrocardiograms of *Drosophila* from the Tub×*W* ^*1118*^‐14d‐N group, the Tub×*W* ^*1118*^‐35d‐N group, the *mtp* ^*RNAi*^‐14d‐N group and *mtp* ^*RNAi*^‐35d‐N, respectively, and quantitatively analyzed for HP, AI, DI, SI, DD, SD, FS, and FL. In comparison with *Drosophila* from the Tub×*W* ^*1118*^‐14d group, Tub×*W* ^*1118*^‐35d‐N group HP, AI, SI, and FL (a, b, d, h) showed a significant increase; DD and FS (e, g) both showed a significant decrease, DI and SD (c, f) did not show a significant difference. Compared with *Drosophila* in the *mtp* ^*RNAi*^‐35d‐N group, *Drosophila* in the *mtp* ^*RNAi*^‐14d‐N group showed an increase in HP, AI, SI, and FL (a, b, d, h) and a decrease in DD (e); DI, SD, and FS (c, f, g) did not show a significant difference. Compared with *Drosophila* in the Tub×*W* ^*1118*^‐14d group, *Drosophila* in the *mtp* ^*RNAi*^‐14d‐N group showed a significant decrease in DD and FS (e, g), and a significant increase in FL (h). Compared with *Drosophila* in the Tub×*W* ^*1118*^‐35d group, *Drosophila* in the *mtp* ^*RNAi*^‐14d‐N group only showed a significant increase in SD (f). (i–l) cardiac cycle maps of *Drosophila* from the Tub×*W* ^*1118*^‐14d‐N group, the Tub×*W* ^*1118*^‐35d‐N group, the *mtp* ^*RNAi*^‐14d‐N group, and the *mtp* ^*RNAi*^‐35d‐N group, respectively, with an M‐mode ECG interception time of 10 s for each group. DD, SD, and FL are marked with a yellow line in the figures. (m) the climbing indices of *Drosophila* in the Tub×*W* ^*1118*^‐14d‐N group, the Tub×*W* ^*1118*^‐35d‐N group, the *mtp* ^*RNAi*^‐14d‐N group, and the *mtp* ^*RNAi*^‐35d‐N group. Climbing ability was significantly reduced in the Tub×*W* ^*1118*^‐35d‐N group compared to the Tub×*W* ^*1118*^‐35d‐N group; compared to the *mtp* ^*RNAi*^‐14d‐N group, climbing ability was also significantly reduced in the *mtp* ^*RNAi*^‐35d‐N group. The climbing ability of *Drosophila* in the *mtp* ^*RNAi*^‐14d‐N group and the Tub×*W* ^*1118*^‐35d group was both significantly lower than that of the Tub×*W* ^*1118*^‐14d group. (n) the *mtp* expression levels of *Drosophila* from 7d to 35d old in the Tub×*W* ^*1118*^ group, and the *mtp* expression levels gradually decreased with age. The sample sizes of the eight groups of *Drosophila* in the M‐mode ECG test were *N* = 17 (Tub×*W* ^*1118*^‐14d‐N), *N* = 17 (Tub×*W* ^*1118*^‐35d‐N), *N* = 18 (*mtp* ^*RNAi*^‐14d‐N), and *N* = 19 (*mtp* ^*RNAi*^‐35d‐N), respectively. The sample size for each group of *Drosophila* in the climbing test was *N* = 100. *mtp* mRNA expression levels were tested in groups with a sample size of *N* = 10. All *Drosophila* were virgin flies. *T*‐tests and ANOVA were used to compare differences between groups. LSD was used for post hoc testing. Data are expressed as mean ± standard deviation (SD). Statistical significance was set at *p* < 0.05.

Cardiac senescence is characterized by a progressive decline in cardiac function, intrinsically associated to the organ itself and correlated with the age of the organism (Ocorr et al., 2007). Compared with *Drosophila* in the Tub×*W* ^*1118*^‐14d‐N group, the Tub×*W* ^*1118*^‐35d‐N group showed a significant decline in cardiac function. Analysis of cardiac rhythms revealed that the heart period, arrhythmia index, and systolic interval were significantly increased, and there was no significant change in the diastolic interval (Figure 1a–d). On the contrary, cardiac systolic function was mainly manifested by a smaller diastolic diameter, a lower fractional shortening, and an increase in fibrillation. By contrast, there was no significant change in the systolic diameter (Figure 1e–j). Previous studies indicated a steady decline in the mean diastolic diameter of wild‐type *Drosophila* hearts over time, with a greater decline compared to systolic diameter, signifying age‐related diastolic dysfunction, as evidenced by a significant reduction in fractional shortening. Our results provide compelling evidence that diastolic performance is significantly diminished in aging (35d) *Drosophila* relative to juvenile (14d) *Drosophila*. This aging‐dependent response suggests impaired myocardial relaxation and possible chamber stiffness, commonly observed during age‐related human diastolic dysfunction. In mammals, aging is characterized by a decline in left ventricular diastolic function, including abnormal diastolic relaxation, chamber filling, and/or passive myocardial stiffness (Kaushik et al., 2012). The heart's pumping and filling properties affect cardiac output and overall cardiac function and can lead to heart failure when dysregulated (Borlaug & Redfield, 2011).

Similar findings were observed in the *mtp* ^*RNAi*^ group. Compared to the *mtp* ^*RNAi*^‐14d‐N group, the *mtp* ^*RNAi*^‐35d‐N group showed significant increases in the heart period, arrhythmia index, systolic interval, and fibrillation and significant decreases in the diastolic diameter; there was no significant change in the diastolic interval, systolic diameter, and fractional shortening (Figure 1a–i,k). Interestingly, after systemic knockdown treatment of *mtp*, the *mtp* ^*RNAi*^‐14d‐N group also showed a significant reduction in cardiac systolic function compared to the Tub×*W* ^*1118*^‐14d‐N group, mainly in the form of a decrease in diastolic diameter and fractional shortening, and an increase in the frequency of fibrillation, while there was no significant difference in systolic diameter (Figure 1e–i,k). We next compared the cardiac systolic function of the Tub×*W* ^*1118*^‐35d‐N group with that of the *mtp* ^*RNAi*^‐14d‐N group and found that, except for a significant difference in the systolic diameter, no significant difference was observed in the indicators of diastolic diameter, fractional shortening, and fibrillation (Figure 1e–h,j,k). On the one hand, along with aging, the decline of cardiac contractile function also affected the climbing ability of *Drosophila*, which was significantly lower in the Tub×*W* ^*1118*^‐35d‐N group than in the Tub×*W* ^*1118*^‐14d‐N group, consistent with findings in the *mtp* ^*RNAi*^‐35d‐N group and *mtp* ^*RNAi*^‐14d‐N group (Figure 1m). On the other hand, the climbing ability of *Drosophila* in the *mtp* ^*RNAi*^‐14d‐N group was significantly lower than that in the Tub×*W* ^*1118*^‐14d‐N group but remained significantly higher than the Tub×*W* ^*1118*^‐35d‐N group (Figure 1m), which may be attributed to the knockdown of *mtp* only affecting the contractile function of the heart, with relatively insignificant effects on other physiological functions related to climbing ability. Meanwhile, the expression level of *mtp* gradually decreased with age and was significantly lower in *Drosophila* in the older group (35d) compared with the younger group (14d) (Figure 1n).

**FIGURE 2:**
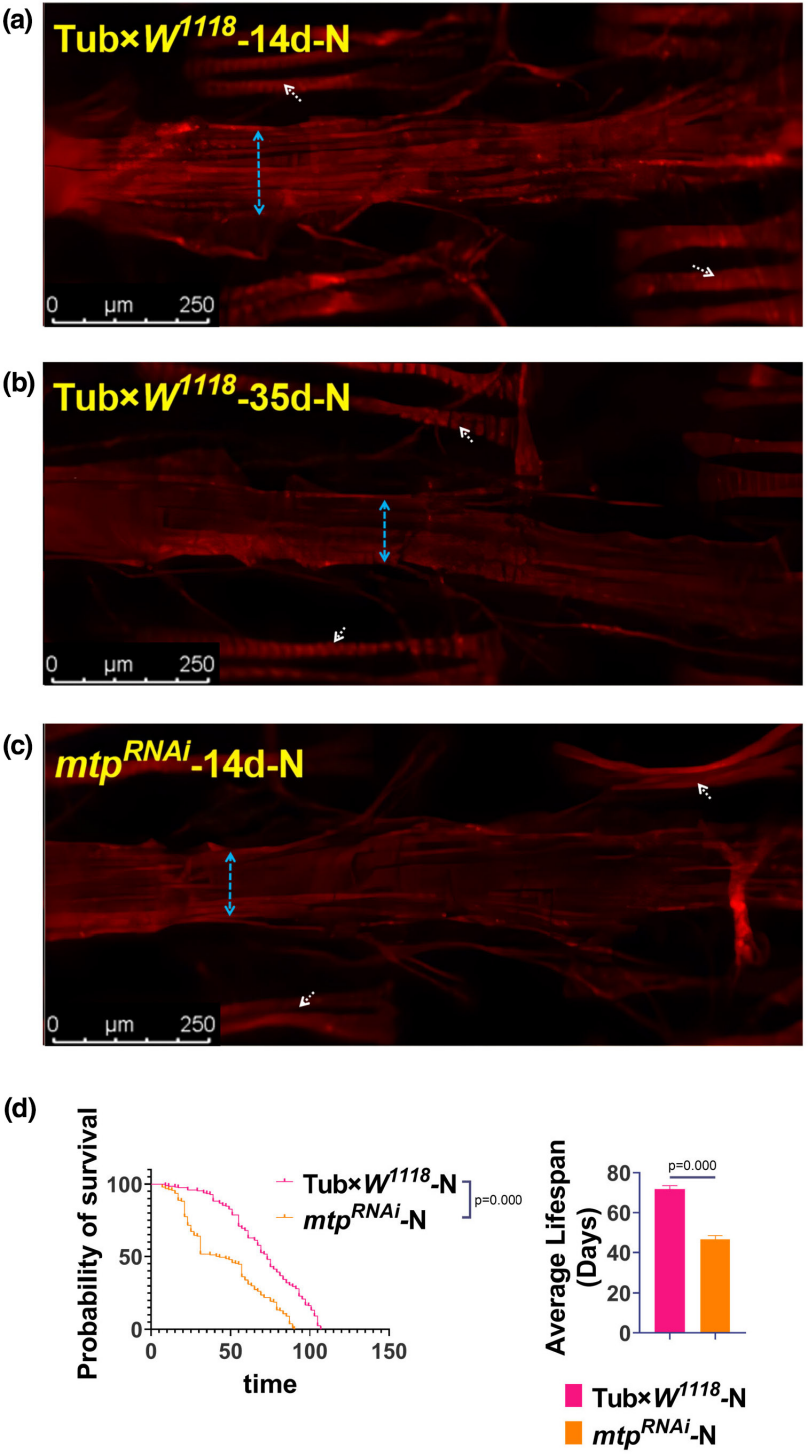
Tub×*W* ^*1118*^‐14d‐N group (a), Tub×*W* ^*1118*^‐35d‐N group (b) and *mtp* ^*RNAi*^‐14d‐N group (c) *Drosophila* cardiac ghost cyclic peptide staining plots; *Drosophila* cardiac ghost cyclic peptide staining plots, *n* = 5. *Drosophila* cardiac tubules were visualized with ghost cyclic peptide‐labeled F‐Actin using a confocal microscope with a scale bar = 250 μm. Blue arrows represent diastolic diameters, and white arrows point to myofiber arrangement. The Tub×*W* ^*1118*^‐35d‐N and *mtp* ^*RNAi*^‐14d‐N groups exhibited a reduction in myocardial diastolic diameter as well as flocculation of myofiber arrangement compared to the Tub×*W* ^*1118*^‐14d‐N group. (d) Survival curves and average lifespan of the Tub×*W* ^*1118*^‐N and *mtp* ^*RNAi*^‐N groups. The sample size of each group was 190–210 *Drosophila*. The average lifespan of *Drosophila* in the Tub×*W* ^*1118*^‐N group was significantly higher than that in the *mtp* ^*RNAi*^‐N group. All samples were virgins. *p*‐Values for the lifespan curves were calculated by log‐rank test. Statistical significance was set at *p* < 0.05.

It is well‐established that the adult *Drosophila* heart resembles a simple linear tube and consists of two major cell types: cardioblasts (CBs), which form the cardiac tube and differentiate into contractile cardiomyocytes, and pericardial cells (PCs), which are irregularly arranged on both sides of the heart and undergo hemolymph filtration. To explore the myofibrillar changes associated with senescence and *mtp* knockdown, we further examined myofibrils in the myocardium, where F‐actin was labeled with ghost pen cyclic peptide. It was found that hearts in the Tub×*W* ^*1118*^‐35d‐N and *mtp* ^*RNAi*^‐14d‐N groups showed a reduction in myocardial diastolic diameter as well as flocculation of myofiber arrangement compared with those in the Tub×*W* ^*1118*^‐14d‐N group (Figure 2a–c). This suggests that both senescence and *mtp* knockdown can impair diastolic function and myofibril structure in *Drosophila* hearts.

Besides, we found that the average lifespan of *Drosophila* in the *mtp* ^*RNAi*^ group was significantly shortened compared with the Tub×*W* ^*1118*^ group (Figure 2d). The changes in the *mtp* ^*RNAi*^‐14d‐N group paralleled the decline in cardiac function and climbing ability due to senescence in *Drosophila* observed in the Tub×*W* ^*1118*^‐35d‐N group, suggesting that there is a close link between the level of *mtp* expression and *Drosophila* cardiac contractile function and lifespan are closely linked.

Our results showed that aging could induce age‐associated diastolic dysfunction in *Drosophila* accompanied by a significant decrease in *mtp* expression levels and systemic knockdown of *mtp* induced premature age‐associated diastolic dysfunction in young *Drosophila* similar to that in old *Drosophila*, suggesting that *mtp* is essential for cardiac systolic function and lifespan in *Drosophila*.

### 3.2 Endurance exercise upregulates the expression of *mtp* to improve cardiac contractile function and extend mean lifespan in aged *Drosophila*

**FIGURE 3:**
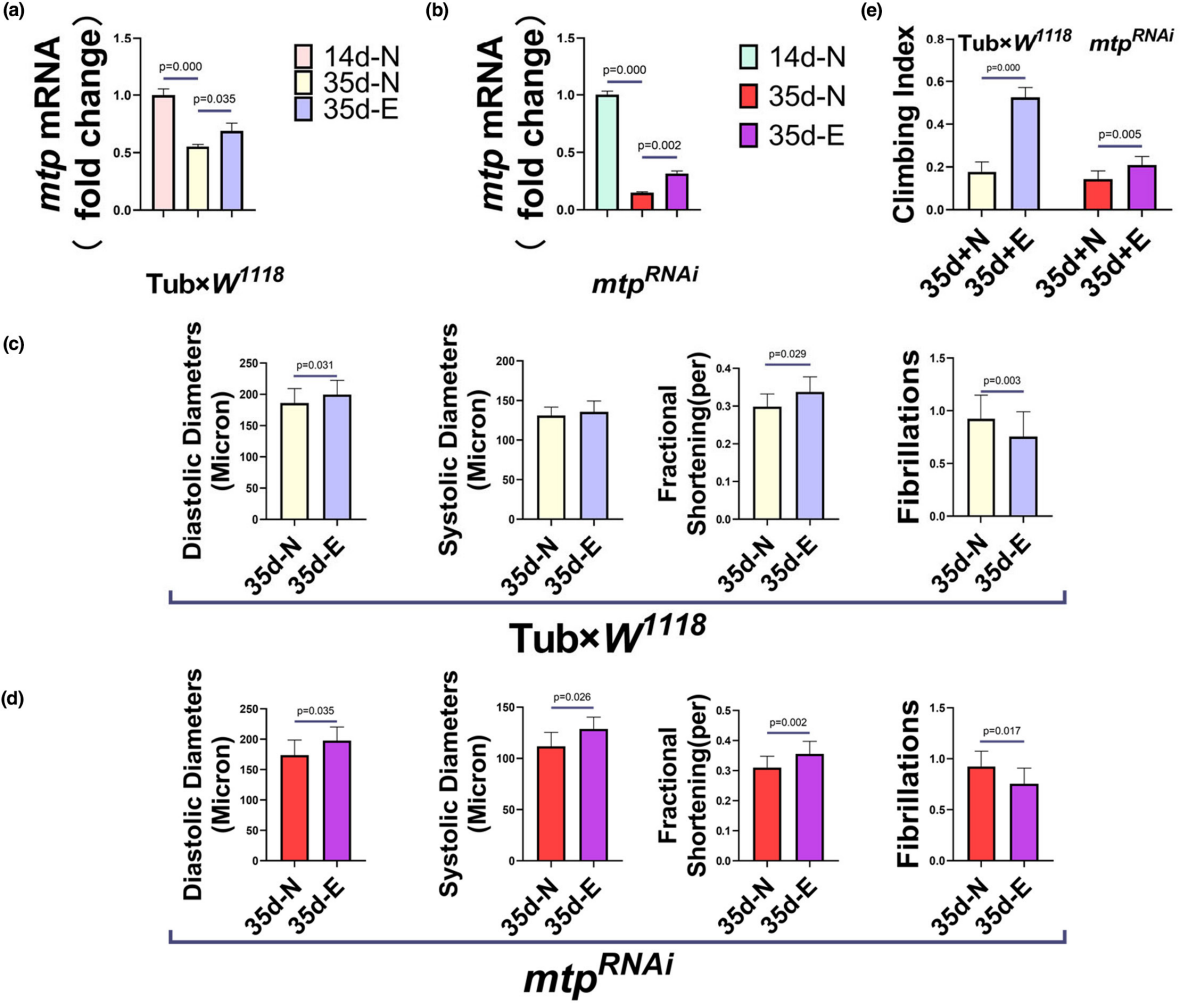
mRNA expression levels of *mtp* in Tub×*W* ^*1118*^ (a) and *mtp* ^*RNAi*^ (b) under different treatments. The mRNA expression level of *mtp* in *Drosophila* in the 35d‐N group of Tub×*W* ^*1118*^ and *mtp* ^*RNAi*^ was significantly decreased compared with the 14d‐N group. The mRNA expression level of *mtp* in *Drosophila* of the 35d‐E group of Tub×*W* ^*1118*^ and *mtp* ^*RNAi*^ increased after 2 weeks of endurance exercise compared to the 35d‐N group. M‐type ECGs and climbing tests were quantitatively analyzed for DD, SD, FS, and FL in *Drosophila* of the 35d‐N and 35d‐E groups of Tub×*W* ^*1118*^ and *mtp* ^*RNAi*^. Quantitative analysis of DD, SD, FS, and FL was performed in the 35d‐E group of Tub×*W* ^*1118*^ and *mtp* ^*RNAi*^: compared to the 35d‐N group, DD and FS appeared to increase and FL decreased significantly in the Tub×*W* ^*1118*^ ‐35d‐E group, and no significant difference was observed in SD (c); compared with the *mtp* ^*RNAi*^‐35d‐N group, DD, SD, and FS appeared to increase in the *mtp* ^*RNAi*^‐35d‐E group, and FL decreased (d). The climbing ability of *Drosophila* in the 35d‐E groups was significantly higher compared with the 35d‐N groups of Tub×*W* ^*1118*^ and *mtp* ^*RNAi*^, and the sample sizes of all four groups of *Drosophila* in the climbing test were *N* = 100 (e). All *Drosophila* were virgin flies, and (a, b) were analyzed using one‐way ANOVA, with LSD used for post hoc testing. All *P* values in (c–e) are from independent samples *t*‐tests. Data are expressed as mean ± standard derivation (SD). Statistical significance was set at *p* < 0.05.

**FIGURE 4:**
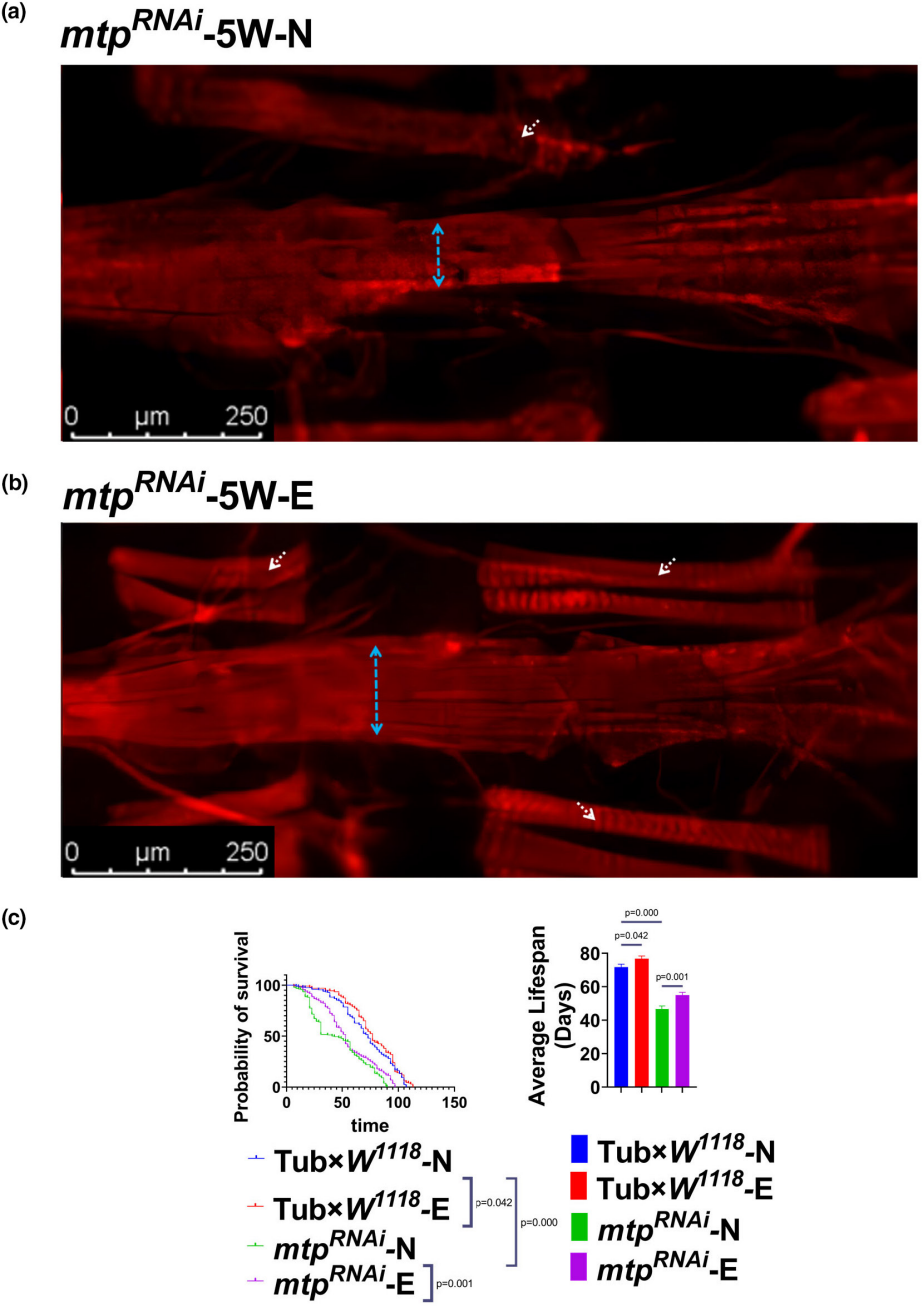
*mtp* ^*RNAi*^ Group 35d‐N group (a) and 35d‐E group (b) Fluorescence images of *Drosophila* hearts, *n* = 5. *Drosophila* cardiac tubes were observed using a confocal microscope with ghost‐pen cyclic peptide‐labeled F‐Actin, scale bar = 250 μm. Blue arrows represent diastolic diameters, and white arrows point to myofiber arrangement. Compared with the *mtp* ^*RNAi*^‐35d‐N group, the *mtp* ^*RNAi*^‐35d‐E group exhibited increased myocardial diastolic diameter and tightly ordered myofibre arrangement. (c) Survival curves and average lifespan of the Tub×*W* ^*1118*^‐N, Tub×*W* ^*1118*^‐E, *mtp* ^*RNAi*^‐N, and *mtp* ^*RNAi*^‐E groups. The sample size of each group was 190–210. The average lifespan of *Drosophila* in the Tub×*W* ^*1118*^‐E group was slightly higher than that in the Tub×*W* ^*1118*^‐N group, and the average life span of *Drosophila* in the *mtp* ^*RNAi*^‐E group was significantly higher than that in the *mtp* ^*RNAi*^‐N group. All samples were virgins. *p* Values for the lifespan curves were calculated by log‐rank test. Statistical significance was set at *p* < 0.05.

Regular moderate‐intensity exercise improves all aspects of human health and is widely used as a preventive and therapeutic strategy for a variety of diseases (Qiu et al., 2023), especially playing an important role in reducing the risk of cardiovascular disease (Cattadori et al., 2018; Channon, 2020; Fiuza‐Luces et al., 2018). The contractile performance of the heart in wild‐type *Drosophila* deteriorates with age, as evidenced by a decrease in diastolic diameter, shortening fraction, and other indices (Kaushik et al., 2012). *Drosophila* from the 35d‐N group of Tub×*W* ^*1118*^ and *mtp* ^*RNAi*^ were found to have significantly higher levels of *mtp* expression after a 2‐week exercise intervention (Figure 3a,b). Correspondingly, the contractile function of *Drosophila* hearts in the Tub×*W* ^*1118*^ and *mtp* ^*RNAi*^'s 35d‐N groups exhibited varying degrees of improvement, which, taken together, reflected an increase of diastolic diameter, an improvement in the shortening fraction as well as a decrease in fibrillation (Figure 3c,d), which indicated that endurance exercise exerted a significant ameliorative effect on *Drosophila*'s aging‐induced diastolic dysfunction (Figure 4a,b). The improvement in cardiac systolic function also affected the climbing ability of *Drosophila*, and the climbing index was significantly improved in both Tub×*W* ^*1118*^ and *mtp* ^*RNAi*^'s 35d‐N groups of *Drosophila* (Figure 3e). In addition, after endurance training, the average lifespan of both the Tub×*W* ^*1118*^ and *mtp* ^*RNAi*^ groups was extended to different degrees, with the *mtp* ^*RNAi*^ ‐E group showing a more significant extension of the average lifespan (Figure 4c). In human studies, regular exercise has been demonstrated to induce systemic adaptations in almost all organ systems, providing multiple health benefits. In this respect, exercise attenuates age‐related multisystem decline and helps maintain physical fitness, including cardiorespiratory fitness, muscle function, flexibility, and balance. For example, in a landmark 21‐year longitudinal study, participation in prolonged running and other vigorous exercise was reported to be associated with less disability and lower mortality in later life in older adults (Chakravarty et al., 2008).

### 3.3 Endurance exercise improves cardiac contractile function and prolongs lifespan in aged *Drosophila* in relation to lipid metabolic pathways downstream of mtp

**FIGURE 5:**
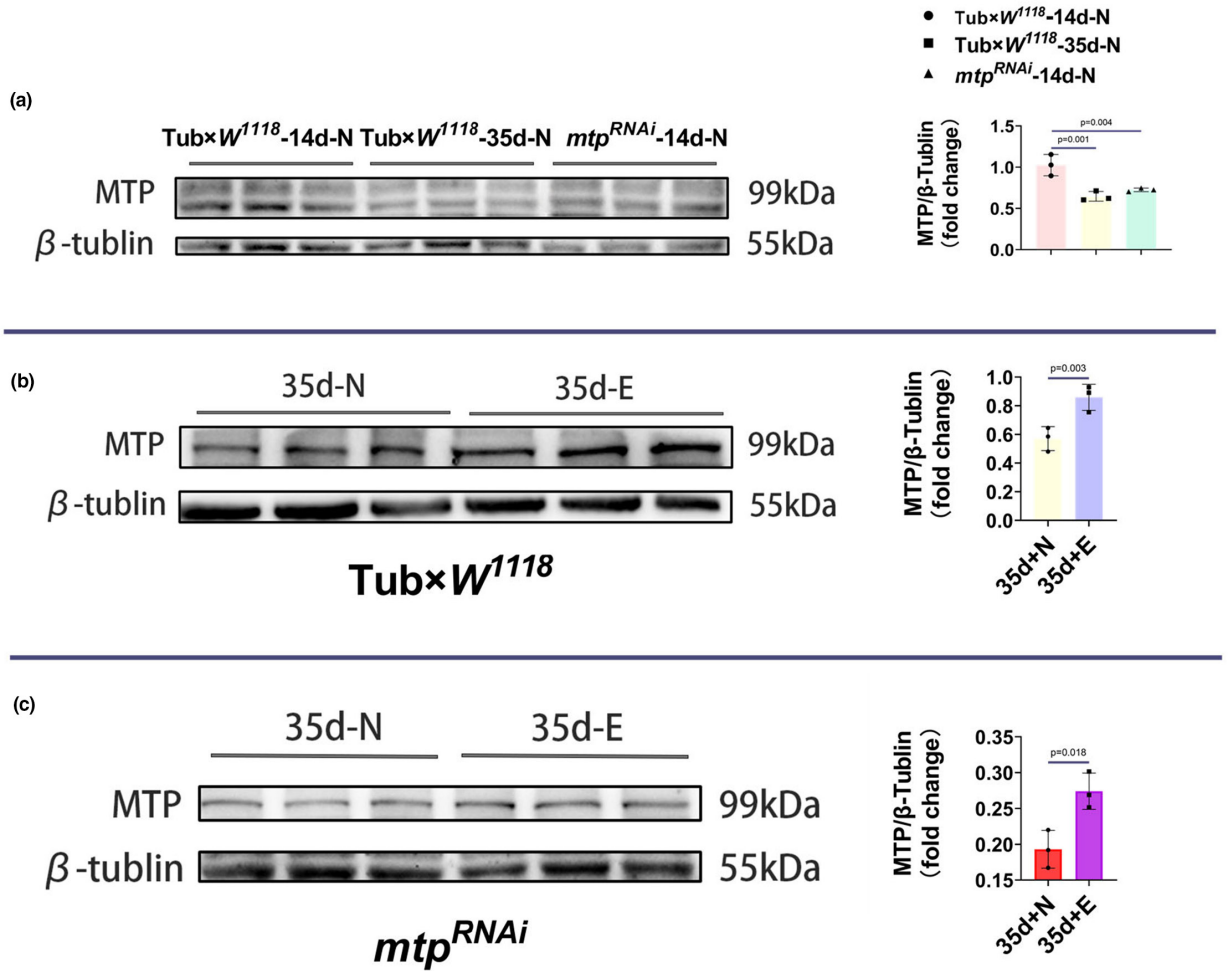
Protein blot analysis using antimicrosomal triglyceride transfer protein and antimicrotubule protein. Representative blots are shown, and the resulting bands were quantified and normalized to microtubule protein levels. (a) MTP levels were significantly higher in the Tub×*W* ^*1118*^‐14d‐N group than in the Tub×*W* ^*1118*^‐35d‐N and *mtp* ^*RNAi*^‐14d‐N groups, and there was no significant difference in MTP levels between the Tub×*W* ^*1118*^‐35d‐N and *mtp* ^*RNAi*^‐14d‐N groups. (b) The MTP content of the Tub×*W* ^*1118*^‐35d‐E group was higher than that of the Tub×*W* ^*1118*^‐35d‐N group. (c) The MTP content in the *mtp* ^*RNAi*^‐35d‐E group was higher than in the *mtp* ^*RNAi*^‐35d‐N group. All samples were virgins, *N* = 10, and measurements were repeated three times. (a) analyzed using ANOVA, and LSD was used for post hoc testing. (b, c) *p* values are from independent samples *t*‐tests. Data are expressed as mean ± standard deviation (SD). Statistical significance was set at *p* < 0.05.

**FIGURE 6:**
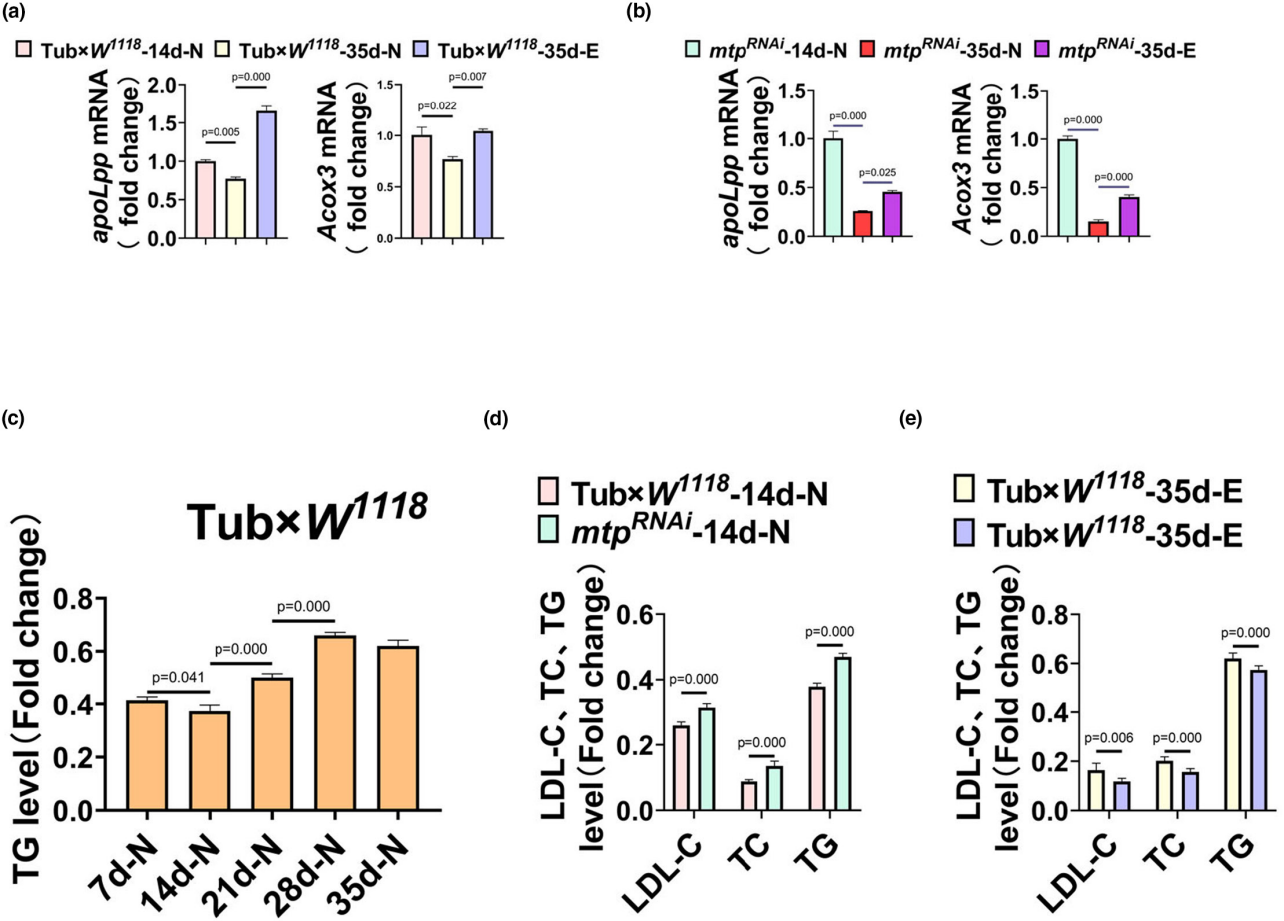
(a) mRNA expression levels of *apolpp* and *Acox3* in 14d‐N, 35d‐N, and 35d‐E groups of Tub×*W* ^*1118*^. The mRNA expression levels of *apolpp* and *Acox3* in *Drosophila* in the 35d‐N group appeared to be reduced compared to the 14d‐N group; the mRNA expression levels of *apolpp* and *Acox3* in *Drosophila* in the 35d‐E group increased significantly after exercise training compared to the 35d‐N. (B) *mtp* ^*RNAi*^ of the mRNA expression levels of *apolpp* and *Acox3* in the 14d‐N, 35d‐N, and 35d‐E groups. The mRNA expression levels of *apolpp* and *Acox3* were significantly reduced in *Drosophila* in the 35d‐N group compared to the 14d‐N group; the mRNA expression levels of *apolpp* and *Acox3* were increased in the 35d‐E group compared to the 35d‐N after exercise training. (C) Whole‐body TG levels in *Drosophila* at different ages in the Tub×*W* ^*1118*^ group. From 14 days of age, the whole‐body TG levels of the Tub×*W* ^*1118*^ group increased significantly with age. (D) LDL‐C, TC, and TG levels in the 14d‐N groups of Tub×*W* ^*1118*^ and *mtp* ^*RNAi*^, and the LDL‐C, TC, and TG levels in the *mtp* ^*RNAi*^‐14d‐N group were significantly higher than those in the Tub×*W* ^*1118*^‐14d‐N group. (E) LDL‐C, TC, and TG levels in the 35d‐N and 35d‐E groups of Tub×*W* ^*1118*^ were significantly lower in the Tub×*W* ^*1118*^‐35d‐E group compared with the Tub×*W* ^*1118*^‐35d‐N group. All samples were virgins, *N* = 10; measurements were repeated three times. (a–c) analyzed using ANOVA, and LSD was used for post hoc testing. (d, e) *p* values are from independent samples *t*‐tests. Data are expressed as mean ± standard deviation (SD). Statistical significance was set at *p* < 0.05.

The mitochondrial triglyceride transfer protein primarily performs a wide range of lipid transport functions required to maintain systemic lipid homeostasis (Shoulders & Shelness, 2005). We hypothesize that endurance exercise upregulates the expression level of *mtp* in aged *Drosophila*, thereby affecting lipid metabolism downstream of *mtp*, leading to improved cardiac contractile function and prolonged lifespan in aged *Drosophila*. In enterocytes and hepatocytes, MTP acts as an endoplasmic reticulum (ER)‐resident protein and is thought to transfer lipids to apolipoprotein B (*apoB*) when *apoB* transcripts are translated, thereby allowing *apoB* to fold correctly and assemble pristine lipoprotein particles (Davidson & Shelness, 2000; Gordon et al., 1995; Hussain, 2000). In addition to this, during the process of β‐oxidation catalyzing the production of acetyl cofactor from long‐chain fatty acids, MTPα, a subunit of MTP, possesses long‐chain 3‐enoyl‐coenzyme A hydratase and long‐chain 3‐hydroxyacyl‐coenzyme A dehydrogenase activities that catalyze the second step (hydration) and the third step (oxidation), respectively, while another subunit, MTPβ, possesses long‐chain 3‐ketoacyl‐coenzyme A thiolase activity that catalyzes the fourth step (sulpholysis). Several epidemiological studies have shown that high levels of LDL‐C and TC are important lipid risk factors for coronary heart disease (Anderson et al., 1987; Gordon et al., 1989; Law et al., 2003; Wetterau et al., 1997). Besides, elevated TG has been documented as an independent risk factor for coronary heart disease (Cui et al., 2001). Therefore, we examined the content of MTP and the mRNA expression levels of *apolpp* and *Acox3* as well as TC, LDL‐C and TG in *Drosophila* from Tub×*W* ^*1118*^ and *mtp* ^*RNAi*^ in the youthful, elderly, and geriatric exercise groups. The content of MTP was significantly reduced in *Drosophila* in the Tub×*W* ^*1118*^‐35d‐N group compared with the 14d‐N group(Figure 5a). Consistently, the expression levels of *apolpp* and *Acox3* were significantly downregulated in *Drosophila* 35d‐N in both the Tub×*W* ^*1118*^ and *mtp* ^*RNAi*^ groups compared with the 14d‐N group (Figure 6a,b), while the content of MTP in both the Tub×*W* ^*1118*^‐35d‐N and *mtp* ^*RNAi*^ ‐14d‐N groups showed no difference (Figure 5a). By contrast, after exercise intervention, the content of MTP and the expression levels of *apolpp* and *Acox3* in the 35d‐E group showed different degrees of elevation compared with the 35d‐N group of Tub×*W* ^*1118*^ and *mtp* ^*RNAi*^ (Figure 5b,c; Figure 6a,b). These trends closely mirrored those of the mRNA expression levels of *mtp* (Figure 3a,b), indicating a significant influence of *mtp* expression on downstream related genes. MTP emerges as a crucial target in the overall lipid metabolic pathway, and its regulation through exercise training holds notable importance. The evaluation of LDL‐C, TC, and TG yielded similar results: in the Tub×*W* ^*1118*^ group, the TG content increased with age from 14 days (Figure 6c), contrary to the declining trend observed in *mtp* expression (Figure 1n). In the *mtp* ^*RNAi*^‐14d‐N group, compared to the Tub×*W* ^*1118*^‐14d‐N group, there was a significant increase in TC, LDL‐C, and TG levels (Figure 6d). The decrease in *mtp* expression compromised *Drosophila*'s lipid metabolism ability, elevating the risk of cardiovascular diseases and reduced climbing ability (Figure 1m). Following the exercise intervention, along with an increase in the expression levels of MTP, *apolpp*, and *Acox3*, the levels of TC, LDL‐C, and TG were reduced (Figure 6e), and the systemic lipid metabolism ability was enhanced, which yielded a protective effect on cardiac function, improved the age‐associated diastolic dysfunction of the aged *Drosophila*, and prolonged lifespan.

## 4 DISCUSSION

Given that cardiovascular disease poses a significant threat to human health, especially with the accelerated aging of society, understanding the progression and control of age‐related changes in cardiac function has become increasingly crucial. Despite this urgency, our knowledge of the genetic mechanisms responsible for the decline in cardiac function with aging remains limited (Cullen, 2000). While endurance exercise improves cardiac function and is used as one of the therapeutic measures for many diseases (Sujkowski et al., 2022), there are no systematic studies on endurance exercise and age‐related diastolic dysfunction, and this knowledge gap suggests that exercise‐dependent molecular mechanisms that could be used to counter age‐related diastolic dysfunction are still largely unknown. Herein, we chose *mtp* as a key target to study the benefits of endurance exercise on age‐related diastolic dysfunction in a precisely controlled and meticulously quantified manner.

In our study, we found that the Tub×*W* ^*1118*^ aged group of *Drosophila* hearts exhibited a marked decline in diastolic diameter (rapid deterioration in diastolic diameter suggestive of age‐related diastolic dysfunction) as well as a significant reduction in shortening fraction, similar to symptoms in wild‐type aged *Drosophila* (Kaushik et al., 2012). Furthermore, an increase in fibrillation was noted, aligning with previous findings. In the mouse heart, *mtp* plays an essential role in myocardial lipid homeostasis, and blocking cardiac *mtp* expression resulted in an approximately 2‐fold increase in myocardial triglyceride stores (Geesaman et al., 2003). Nielsen and colleagues found that in patients with coronary artery disease, left ventricular *mtp* mRNA levels were negatively correlated with tissue triglyceride content, and it was hypothesized that upregulation of transcripts of *mtp* (and possibly *apoB*) in the ventricle could contribute to the reduction of triglyceride accumulation in hypoxic hearts (Bjorkegren et al., 2001). The West of Scotland Coronary Prevention Study (WOSCOPS) found that the *mtp*‐ 493 t allele or linked imbalanced alleles could increase the risk of coronary events by a mechanism associated with reduced cardiac *mtp* expression (Nielsen et al., 2002). In the present study, systemic inhibition of *mtp* revealed that the young group of *mtp* ^*RNAi*^ showed a significant increase in TC, LDL‐C, and TG and would exhibit diastolic dysfunction similar to that of aged Tub×*W* ^*1118*^, as well as a substantial shortening of the average lifespan of *mtp* ^*RNAi*^ group, which suggests that *mtp* not only influences systemic lipid metabolism but also may be associated with the age‐related decline in cardiac function as well as lifespan. Since the subunits of *mtp* are involved in the β‐oxidation of fatty acids, there may be a correlation between changes in *mtp* and mitochondrial alterations. mRNA expression levels of *mtp* may affect OXPHOS genes, mitochondrial fusion/fission, and mitochondrial phagocytosis genes (Parra et al., 2014), leading to age‐related changes in mitochondria, which may ultimately affect lifespan, emphasizing the need for more research.

Exercise therapy is a cost‐effective therapy to reduce the risk of developing cardiovascular disease and mortality (Goenka & Lee, 2017). In aged *Drosophila*, exercise enhances cardiac function and improves cardiomyocyte ultrastructure and heart failure (Piazza et al., 2009). The CG9940 gene encodes a NAD(+) synthase protein in *Drosophila*. Low expression of CG9940 negatively affects lifespan, and exercise slows down the age‐related declines in cardiac function, mobility, and lifespan of these flies (Wen et al., 2016). Similar results were found in our study, where endurance exercise significantly improved cardiac systolic function, especially diastolic diameter and shortening fraction, in aged *Drosophila* and acted as a good therapeutic agent for age‐related diastolic dysfunction. In our previous study, exercise intervention was able to upregulate the cardiac expression of *mtp* and improve cardiac dysfunction and lipid metabolism abnormalities in high‐fat *Drosophila* (Peng et al., 2022). Meanwhile, we found that the climbing ability and average lifespan of old *Drosophila* increased, especially in the *mtp* ^*RNAi*^ group. This suggests that *mtp* may be an important target for exercise training to ameliorate age‐associated diastolic dysfunction and extend lifespan. Related studies have shown that exercise activates the cardiac dSir2/Foxo/SOD and dSir2/Foxo/bmm pathways and reduces the occurrence of diastolic dysfunction as well as enhances cardiac contractility (Rosenberg & Parkhurst, 2002; Wen et al., 2019). Based on our findings, it can be inferred that the regulatory pathways of mtp are complex and numerous, with the insulin pathway (Hagan et al., 1994), cholesterol (Sato et al., 1999), bile acids (Hirokane et al., 2004), endotoxin (LPS), cytokines tumor necrosis factor (TNF), IL‐1, and IL‐6 (Navasa et al., 1998), among others, all playing a role in the regulation of mtp. For example, in HepG2 cells, insulin inhibits *mtp* promoter activity in a dose‐ and time‐dependent manner, and insulin inhibits *mtp* transcription through activation of the MAPKerk cascade rather than through the phosphatidylinositol 3‐kinase pathway (Hagan et al., 1994). On the contrary, *mtp* promoter activity appears to be positively regulated by cholesterol in a dose‐dependent manner, and cholesterol regulation of *mtp* promoter activity is not dependent on the trans‐activating structural domain of the SREBP protein. Instead, the SRE‐binding protein (SREBP) binds to the ‐124 ‐116 SRE site and thus negatively regulates *mtp* gene expression (Sato et al., 1999). A clear relationship has been established between endurance exercise and the insulin pathway as well as cholesterol, and it is highly conceivable that the expression level of *mtp* may be indirectly regulated through the insulin pathway, cholesterol, or other molecular pathways, which will require more in‐depth studies in the future.

Studies on rats suggested that microvascular dysfunction and insufficient coronary perfusion could be the mechanism underlying diastolic dysfunction in aged rats (Hotta et al., 2017). In our study, we found that the decrease in mRNA and protein levels of *mtp* due to aging could impact systemic lipid metabolism, and the mRNA expression levels of *apolpp* and *Acox3* downstream of *mtp* could be decreased, while the levels of TC, LDL‐C, and TG could be significantly increased. Dysregulation of lipid metabolism could lead to impaired cardiac function, with decreases in diastolic diameter and shortening fraction accompanied by an increase in fibrillation, which may account for the early onset of age‐related diastolic dysfunction in *Drosophila* in the *mtp* ^*RNAi*^ youth group. For example, individuals with higher levels of HDL (good cholesterol) and lower levels of LDL (bad cholesterol) have a relatively lower risk of heart disease and stroke compared to their peers (Barzilai et al., 2001; Terry et al., 2003).

In subjects without cardiovascular disease, regular physical activity reduces the risk of cardiovascular death by 30% (Nocon et al., 2008; Paffenbarger Jr et al., 1986), and adequate exercise leads to an additional 1–2 years of life compared to those with little or no physical activity (Franco et al., 2005; Kent‐Braun et al., 2000). Longevity studies have examined apolipoprotein E (ApoE), a gene involved in the metabolism of lipoproteins, and ApoE E3 and E2 have been associated with a lower risk of death in individuals who are regularly physically active compared to sedentary individuals (Dankner et al., 2020). After endurance exercise, *Drosophila* in the exercise group showed increased levels of *mtp* expression and showed varying degrees of prolongation of mean and maximum lifespan compared to *Drosophila* without exercise intervention. Lipid metabolism is crucial in regulating aging and longevity (Mutlu et al., 2021). According to our understanding, the prolonged lifespan may be attributed to the substantial mitigation of age‐induced declines in both mRNA and protein levels of *mtp* through endurance exercise. This effect, in turn, leads to increased mRNA expression levels of *apolpp* and *Acox3*, resulting in decreased levels of TC, LDL‐C, and TG. Consequently, there is an enhancement in whole‐body lipid metabolism, promoting an increase in systolic diameter and shortening fraction. This improvement in cardiac function contributes to reduced lipid accumulation in aging flies, ultimately serving as a mechanism to ameliorate age‐related diastolic dysfunction and extend the average lifespan. Our results can be summarized as follows: (1) the *mtp* ^*RNAi*^ youth group of *Drosophila* exhibits age‐related diastolic dysfunction similar to that of the Tub×*W* ^*1118*^ age group and has a significantly shorter lifespan, which may be related to the low level of *mtp* expression. (2) Endurance exercise improves diastolic dysfunction and prolongs lifespan in old *Drosophila*, which may be related to the upregulation of *mtp* expression by endurance exercise. (3) Relevant assays of lipid metabolism showed that compared with the Tub×*W* ^*1118*^ youth group, the *mtp* ^*RNAi*^ youth group exhibited higher TC, LDL‐C, and TG levels, while endurance exercise could reduce the TC, LDL‐C, and TG levels in the aged group, which may be related to the increased expression of *mtp* downstream of *apolpp* and *Acox3*.

## 5 CONCLUSION

**FIGURE 7:**
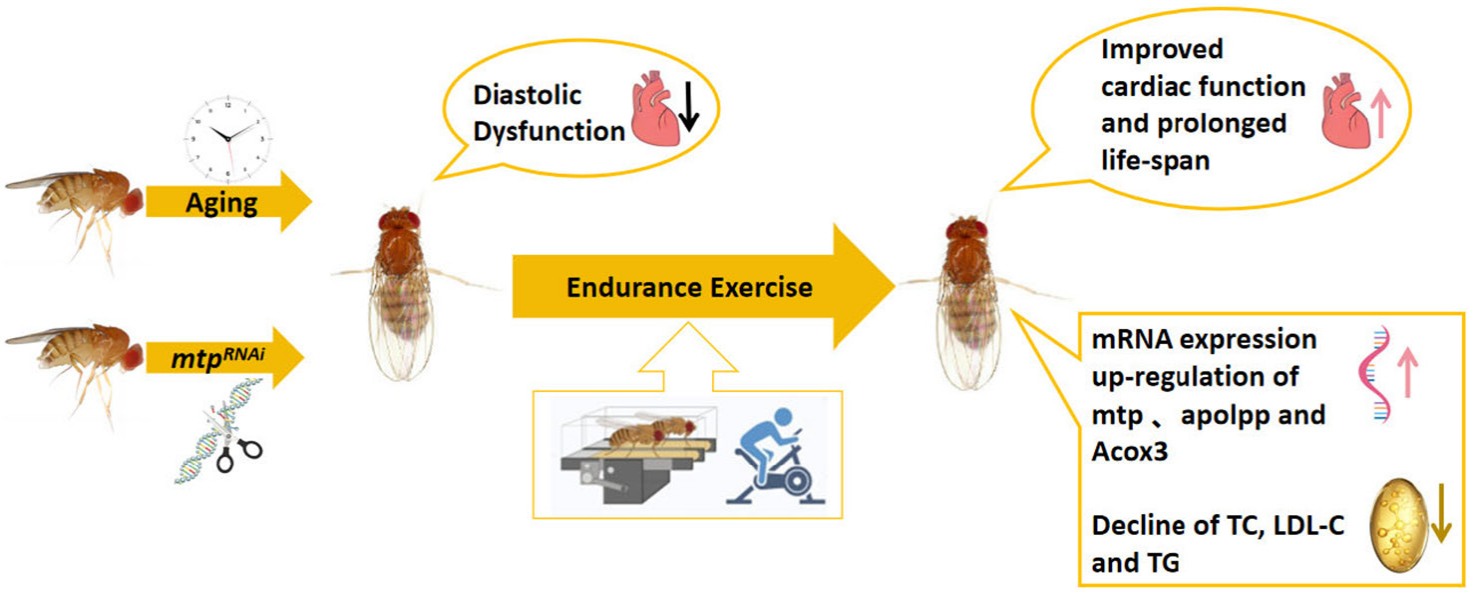
Schematic diagram of the relationship between endurance exercise, *mtp*, and age‐related diastolic dysfunction.

Our findings suggest that endurance exercise ameliorates age‐related diastolic dysfunction and prolongs average lifespan, which may be related to the upregulation of *mtp* expression and thus enhances lipid metabolism in aged *Drosophila* (Figure 7). Importantly, our findings provide new insights for future research on cardiac aging and exercise interventions to improve age‐related diastolic dysfunction and prolong lifespan.

## AUTHOR CONTRIBUTIONS

Tianhang Peng was involved in conceptualisation and writing—original draft preparation. Tianhang Peng and Meng Ding were involved in methodology. Hanhui Yan, Ping Zhang and Rui Tian were involved in formal analysis and investigation. Yin Guo and Lan Zheng were involved in writing—review and editing. Lan Zheng was involved in funding acquisition, resources, and supervision. All authors have read and approved the final manuscript.

## FUNDING INFORMATION

This research was funded by the National Natural Science Foundation of China, grant number 32071175.

## CONFLICT OF INTEREST STATEMENT

The authors declare no conflict of interest.

## ETHICS STATEMENT

Not applicable.

## Data Availability

Data are contained within the article.
